# Fecal Viral Concentration and Diarrhea in Norovirus Gastroenteritis

**DOI:** 10.3201/eid1309.061535

**Published:** 2007-09

**Authors:** Nelson Lee, Martin C.W. Chan, Bonnie Wong, K.W. Choi, Winnie Sin, Grace Lui, Paul K.S. Chan, Raymond W.M. Lai, C.S. Cockram, Joseph J.Y. Sung, Wai K. Leung

**Affiliations:** * Prince of Wales Hospital, Hong Kong Special Administrative Region, People’s Republic of China

**Keywords:** Norovirus, viral concentration, diarrhea, gastroenteritis, Hong Kong, dispatch

## Abstract

Fecal viral concentrations of 40 patients infected with norovirus genogroup GII.4 correlated with diarrhea duration and frequency of vomiting. Higher viral concentration and older age were independently associated with prolonged diarrhea (>4 days). These findings provide information on the pathogenesis and transmission of norovirus infections.

Norovirus is a major cause of viral gastroenteritis worldwide, accounting for at least 28% of all foodborne outbreaks (*1*). However, its pathogenesis is poorly understood (*2*). Although the disease is usually perceived as mild and self-limiting (symptoms generally subside within 2–3 days in otherwise healthy persons) (*1*,*2*), protracted diarrhea and serious complications may develop in elderly or immunocompromised patients (*2*–*4*).

We have previously shown that patients infected with norovirus genogroup GII have at least 100-fold higher fecal viral concentrations than those infected with genogroup GI (*5*), which may help explain the former’s global predominance (*6*,*7*). However, whether fecal viral concentration has any association with disease manifestation is unknown. In this study, we postulated that a higher viral concentration is associated with more severe symptoms. We studied potential associations in patients infected with norovirus GII.4, the predominant norovirus genotype circulating in Hong Kong during the study period (*6*,*7*).

## The Study

During a 2-year period (November 2004–November 2006), 44 adult (>16 years of age) patients at 2 regional hospitals in Hong Kong Special Administrative Region with acute gastroenteritis were shown to be infected with norovirus genogroup GII.4. Clinical records were reviewed and baseline characteristics, clinical features, and output charts were studied. Cases were included for analysis if stool samples were collected <96 hours from symptom onset. Diarrhea was defined as having >3 loose stools per day. Duration of diarrhea was defined as the number of days (inclusive) between the first and final dates of symptoms (*3*).

Stool samples were collected from all patients when initially observed and processed immediately for RNA extraction. Diagnosis of norovirus infection and its quantitation were based on real-time reverse transcription–PCR assay of stool samples as described (*5*). The lower detection limit of the assay was 2 × 10^4^ copies of cDNA/g stool. Phylogenetic studies were also performed as described (*5*).

Associations between clinical parameters and fecal viral cDNA concentrations were determined. Univariate associations between fecal viral concentration (log_10_ copies cDNA/g fecal specimen), baseline characteristics, and clinical variables were examined by using the Mann-Whitney test or χ^2^ test as appropriate. Variables with a p value <0.1 in univariate analyses were entered into multivariate models as covariates. Stepwise backward logistic regression was performed to identify independent variables associated with prolonged diarrhea, defined as >4 days of diarrhea. This cut-off was based on the results of many observational studies (*1*–*4*) and was also above the median duration of diarrhea in this cohort. Spearman rank correlation coefficient (ρ) was used to assess correlations between viral cDNA concentration and other continuous variables. A p value <0.05 was considered statistically significant. All probabilities were 2-tailed. Statistical analysis was performed with SPSS software version 13.0 (SPSS Inc., Chicago, IL, USA).

Stool samples from 40 patients were analyzed. Mean ± SD age of patients was 60.4 ± 24.3 years and 15 (37.5%) were males. Seventeen patients (42.5%) had pre-existing medical conditions, and 21 (52.5%) were hospitalized. Diarrhea was observed in 100%, whereas vomiting and fever were observed in 64.9% and 36.8%, respectively. Median duration of diarrhea was 3 days (range 2–6 days). Median fecal cDNA concentration was 8.93 log_10_ copies/g stool (interquartile range 8.22–10.24 log_10_ copies/g stool).

**Figure 1:**
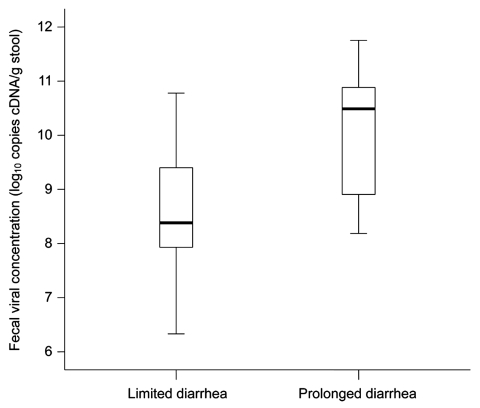
Box plot of median (black horizontal bars) and interquartile range (error bars) of fecal norovirus cDNA concentrations in patients with limited and prolonged diarrhea. Limited diarrhea is defined as a total duration of diarrhea of 1–3 days, and prolonged diarrhea is defined as a total duration of diarrhea ≥4 days.

Fecal viral cDNA concentration was examined in relation to baseline characteristics and clinical symptoms (Table). Higher viral concentrations were associated with older age (p = 0.064). Higher fecal viral concentration was significantly associated with prolonged diarrhea >4 days (2.11 log_10_ copies/g stool; p = 0.001, by Mann-Whitney test) than with limited diarrhea (Figure 1). Viral concentration was positively correlated with total duration of diarrhea (Spearman ρ 0.47, p = 0.004) and total frequency of vomiting (Spearman ρ 0.34, p = 0.043) during the course of illness (Appendix Figure). Fever developed more frequently in patients with prolonged diarrhea (64.3% vs. 21.7%; p = 0.010, by χ^2^ test). Mean total frequency of diarrhea and vomiting was 14.9 and 3.1, respectively, in inpatients with prolonged diarrhea and 11.8 and 1.2, respectively, in those with limited diarrhea. We did not observe an association between mean daily output and fecal viral concentrations in this cohort.

**Table:** Fecal viral concentrations of 40 patients infected with norovirus*

Comparison groups (%)	Median fecal viral concentration, log_10_ copies cDNA/g stool (IQR)	p value
Age, y		
<65 (47.5)	8.48 (7.79–10.11)	0.064
>65 (52.5)	8.97 (8.54–10.70)	
Sex		
Male (37.5)	8.97 (7.79–10.72)	0.706
Female (62.5)	8.88 (8.24–10.22)	
Pre-existing medical conditions†		
No (57.5)	8.95 (7.86–10.26)	0.520
Yes (42.5)	8.91 (8.31–10.14)	
Diarrhea duration‡		
Limited (62.2)	8.38 (7.89–9.45)	0.001
Prolonged (37.8)	10.49 (8.84–10.94)	
Vomiting		
No (35.1)	8.71 (7.71–9.91)	0.215
Yes (64.9)	9.10 (8.25–10.40)	
Fever§		
No (62.2)	8.77 (8.15–10.17)	0.380
Yes (36.8)	9.13 (8.23–10.75)	

*IQR, interquartile range. †Includes diabetes mellitus, chronic cardiovascular/pulmonary/hepatic diseases, and underlying malignancies. No patient had conditions associated with profound immunosuppression in this cohort. ‡Limited (62.2%) is defined as a total duration of diarrhea of 1–3 d, including both hospitalized and nonhospitalized patients (followed up by the same hospital’s emergency departments as outpatients).Prolonged (37.8%) is defined as a total duration of diarrhea >4 d; all but 1 were hospitalized patients. §Temperature >37.5°C on >1 occasion.

**Figure 2:**
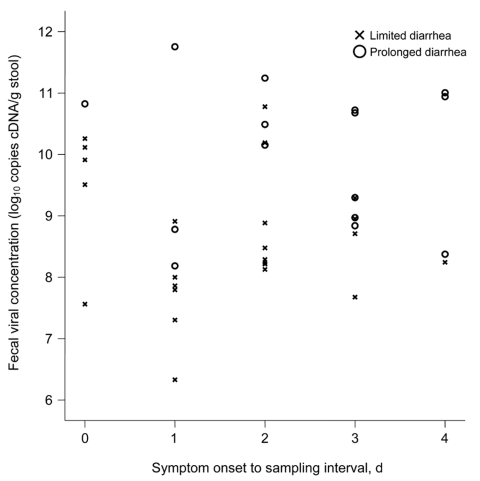
Scatter plot showing fecal norovirus cDNA concentrations in patients with limited and prolonged diarrhea, according to sampling day. Limited diarrhea is defined as a total duration of diarrhea of 1–3 days, and prolonged diarrhea is defined as a total duration of diarrhea >4 days.

To rule out possible confounding by variations in sample collection time, fecal viral cDNA concentration was also examined by sample collection day (Figure 2). In general, samples collected from patients with prolonged diarrhea had higher viral concentrations on all collection days. The mean day of sample collection was slightly later in patients with prolonged diarrhea than in those with limited symptoms (2.4 ± 1.3 days vs. 1.5 ± 1.1 days; p = 0.045, by *t* test).

Prolonged diarrhea >4 days was associated with older age and pre-existing medical conditions by univariate analyses (p<0.05 for both variables, by χ^2^ test) and with fecal viral concentration. Stepwise backward logistic regression analysis showed that fecal viral concentration (odds ratio [OR] 9.56, 95% confidence interval [CI] 1.18–77.57 per log_10_ copies; p = 0.035) and age (OR 1.15, 95% CI 1.03–1.28) per year; p = 0.013) were 2 independent factors associated with prolonged diarrhea caused by norovirus genotype GII.4.

## Conclusions

To our knowledge, this is the first clinical study to demonstrate that fecal viral concentration correlates with duration of illness in norovirus gastroenteritis. It has been reported that severe protracted diarrhea caused by norovirus infection can develop in hospitalized, elderly, and immunocompromised patients (*3*,*4*,*8*). Such patients often shed virus for prolonged periods, which probably indicates active viral replication and slow viral clearance (*4*,*8*). In an animal model, norovirus was shown to infect and possibly replicate in enterocytes, resulting in disease (*9*). Results of our study thus provide preliminary evidence that active viral replication determines clinical disease in norovirus gastroenteritis, as in most other viral infections (*10*). These findings also suggest that more stringent infection control measures need to be implemented in patients with severe diarrhea because of high fecal viral concentrations (*1*–*4*,*8*).

This study was limited by a small sample size and fecal viral concentration, which was studied only at 1 time point for each patient. Further research on changes in fecal viral concentrations and their relationships with disease severity are warranted. Because no previous clinical studies describe temporal changes of norovirus concentration in relation to symptoms, we analyzed viral concentration data only in patients with acute diarrhea (days 0–4). Although our definition of prolonged diarrhea (>4 days, which was above the median in our cohort) seemed arbitrary, it is supported by the results of many observational studies, which show that in most patients (even elderly or hospitalized patients), acute symptoms subside within 2–3 days (*1*–*4*,*8*). Inclusion of only norovirus GII.4 infections in the analysis removed the possible confounder of strain variation on viral concentration (*5*). Whether similar correlations can be observed with other norovirus strains remains uncertain. Given that genogroup GII.4 is the predominant circulating strain in most countries with major outbreaks (*6*), these results have implications with regard to pathogenesis and infection control of norovirus infections.

In conclusion, these results provide preliminary evidence that a high fecal viral concentration is independently associated with prolonged norovirus gastroenteritis. Further studies are needed to confirm the role of enhanced viral replication on pathogenesis and transmission of this disease. In addition, the approach of quantifying norovirus by real-time PCR can be used for future evaluation of antiviral treatment (*11*) and to study factors associated with delayed viral clearance (*3*).

## Supplementary Material

Appendix Figure A) Fecal viral concentrations (log10 copies cDNA/g stool) plotted against duration of diarrhea (d). B) Fecal viral concentrations (log10 copies cDNA/g stool) plotted versus total frequency of vomiting. Black horizontal bars show medians, and error bars show interquartile ranges.
