# Supplementary figures and images for: Fecal Viral Concentration and Diarrhea in Norovirus Gastroenteritis
Source: Emerg Infect Dis. 2007 Sep;13(9):1399–401. doi: 10.3201/eid1309.061535 (PMC2857278; doi:10.3201/eid1309.061535)

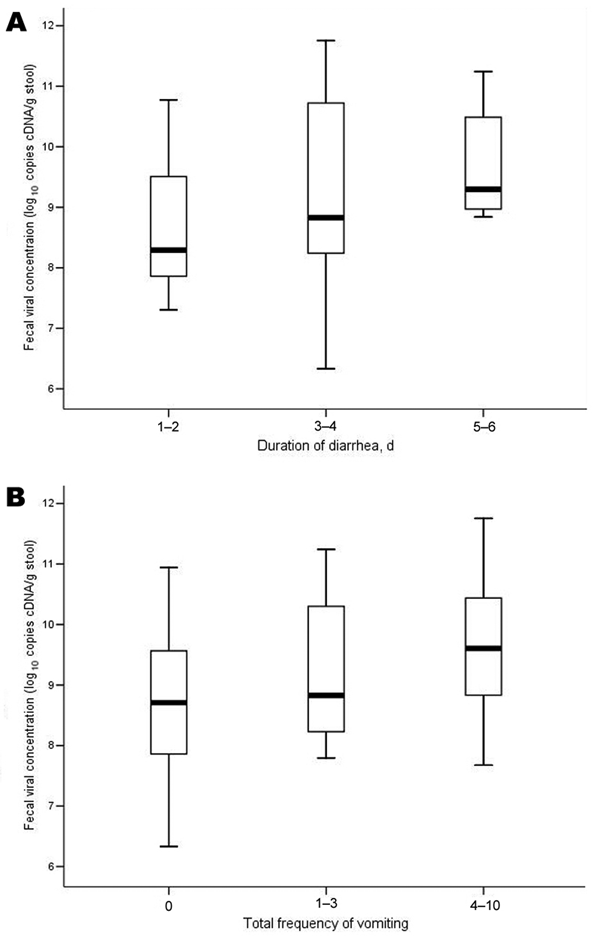

Supplement: Appendix Figure — A) Fecal viral concentrations (log10 copies cDNA/g stool) plotted against duration of diarrhea (d). B) Fecal viral concentrations (log10 copies cDNA/g stool) plotted versus total frequency of vomiting. Black horizontal bars show medians, and error bars show interquartile ranges. [file 06-1535_appF-s1.gif]
